# The protective efficacy of omega-3 polyunsaturated fatty acids on oxidative stress, inflammation, neurotransmitter perturbations, and apoptosis induced by monosodium glutamate in the brain of male rats

**DOI:** 10.1007/s11011-025-01539-4

**Published:** 2025-01-29

**Authors:** Amina E. Essawy, Eman M. Jimmiey, Wessam M. Abdel-Wahab, Rania G. Ali, Saber M. Eweda, Heba M. Abdou

**Affiliations:** 1 Department of Zoology, Faculty of Science, Alexandria University, Alexandria, 21515 Egypt; 2 Department of Pathology, Faculty of Medicine, Alexandria University, Alexandria, Egypt; 3 Department of Biochemistry, Faculty of Science, Alexandria University, Alexandria, 21515 Egypt; 4 Department of Clinical Laboratories Sciences, College of Applied Medical Sciences, Taibah University, Madinah, 42353 Kingdom of Saudi Arabia

**Keywords:** Monosodium glutamate, Omega-3 polyunsaturated fatty acids, Oxidative stress, Neuroinflammation, Apoptosis

## Abstract

Exaggerated neuronal excitation by glutamate is a well-known cause of excitotoxicity, a key factor in numerous neurodegenerative disorders. This study examined the neurotoxic effect of monosodium glutamate (MSG) in the brain cortex of rats and focused on assessing the potential neuroprotective effects of omega-3 polyunsaturated fatty acids (ω-3 PUFAs). Four groups of adult male rats (*n* = 10) were assigned as follows; normal control, ω-3 PUFAs (400 mg/kg) alone, MSG (4 mg/g) alone, and MSG plus ω-3 PUFAs (4 mg/g MSG plus 400 mg/kg ω-3 PUFAs). Biochemical analysis, immunohistochemical, and histological examinations were conducted upon completion of the treatment protocol. Results revealed that MSG significantly increased malondialdehyde, nitric oxide, tumor necrosis factor-α, interleukin 1β, acetylcholinesterase, monoamine oxidase, and caspase-3. However, the MSG-treated group showed a decline in reduced glutathione, catalase, superoxide dismutase, dopamine, and serotonin. In addition, MSG caused histopathological changes in the cortical region which support the biochemical and immunohistochemical analysis. Supplementation of ω-3 PUFAs greatly improved the biochemical, immunohistochemical, and histopathological alterations induced by MSG administration in the brain cortex. Together, these findings revealed a neuroprotective effect of ω-3 PUFAs against MSG-induced toxicity in the brain cortex by attenuating oxidative damage, inflammation, neurochemical perturbations, and apoptosis.

**Graphical abstract:**

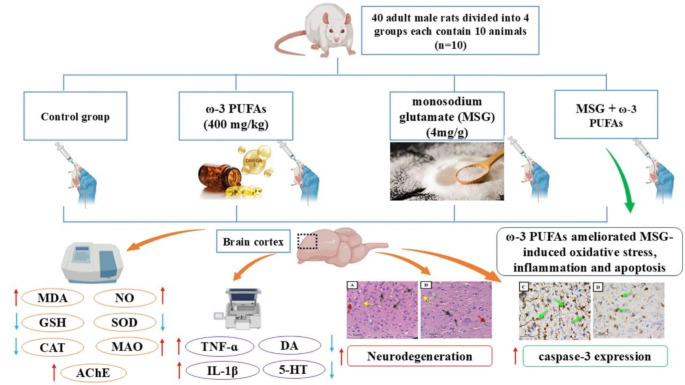

## Introduction

Glutamate is a nonessential amino acid that can be produced in the human body in amounts adequate for its use (Sahin et al. 2023). The naturally occurring glutamic acid in food does not affect metabolism, whereas, synthetic glutamic acid such as monosodium glutamate (MSG) might have toxic effects (Chakraborty 2019). MSG is a food additive widely used as a flavor enhancer with a meaty and savory flavor (Thuy et al. 2020). The breakdown of MSG in processed food leads to the release of free glutamate, causing a rise in extracellular glutamate levels (Abu-Elfotuh et al. 2022). Even though the US Food and Drug Administration has classified MSG as a food ingredient generally identified as safe when used within limited concentrations, there is ongoing debate about the safety of MSG (Maluly et al. 2017). Several studies on humans and experimental animals reported that prolonged consumption of MSG has deleterious effects on various organs such as the liver, kidney, thymus, pancreas, testis, and brain (Hajihasani et al. 2020; Hamza and Diab 2020). Furthermore, excessive intake of MSG can increase the incidence of some metabolic disorders such as type 2 diabetes mellitus, obesity, and reproductive disorders (Hernández-Bautista et al. 2014; Niaz et al. 2018; Banerjee et al. 2021). Glutamate is utilized as the primary excitatory neurotransmitter in about 75% of synapses in the central nervous system for intercellular communication (Teleanu et al. 2022). Increased extracellular glutamate causes aberrant synaptic signaling leading to neuronal excitotoxicity, often causing nerve cell death due to excessive activation of glutamate receptors (Iovino et al. 2020). Studies on MSG in different areas of the brain have consistently shown its harmful effect on neurotransmission, cognition, and behavior (Gbadamosi et al. 2024). Continued high levels of glutamate in the brain can result in neurological toxicity and increase the chances of developing neurodegenerative conditions such as Parkinson’s and Alzheimer’s diseases (Ankul et al. 2023; Ko et al. 2022). Cholinergic dysfunction, oxidative, apoptotic, and inflammatory challenges are important contributors to the neurotoxic effects of MSG (Sadek et al. 2016; Albrakati 2022). It is noteworthy that excitotoxicity, neuroinflammation, and oxidative stress have the ability to continue to feed one another, allowing this “neurotoxic triad” to be maintained. One of the well documented key hallmarks in the neurotoxicity of MSG is the oxidative stress which can easily affect the brain due to its high metabolic activity with low antioxidant capacity. In addition to causing cell damage and apoptosis, free radicals can activate signaling pathways of inflammation and may disrupt the blood-brain barrier (Song et al. 2020).

The use of dietary supplements can help prevent or lower the harmful effects caused by substances. The varied biological and therapeutic effects of omega-3 polyunsaturated fatty acids (ω−3 PUFAs) have sparked significant interest in their beneficial health impacts (Oktar et al. 2024; Xia et al. 2024). These essential fatty acids are derived mainly from fish and seafood and include α-linolenic acid, stearidonic acid, eicosapentaenoic acid (EPA), and docosahexaenoic acid (DHA) (Prasad et al. 2021). They have been reported to exert anti-inflammatory, antihypertensive, anti-lipidemic, anti-thrombotic, and vasodilatory actions (Jump et al. 2012). In eight weeks, older individuals showed increased insulin sensitivity due to the active metabolites in diets rich in ω−3 PUFAs supplementation (Sinha et al. 2023). Moreover, the anticarcinogenic effect of ω−3 PUFAs against various types of cancer including, lung, colorectal, pancreatic, and prostate has been reported (D’Eliseo and Velotti 2016). Dietary fats containing ω−3 PUFAs are necessary for maintaining the fluidity of neuronal membranes and regulating the neurotransmitters essential for proper brain function (Stachowicz 2023). Increased ω−3 PUFAs intake has lowered the risk of dementia, particularly in Alzheimer’s disease, as they are essential constituents of membrane phospholipids in the brain (Wood et al. 2022). Consuming fish oil rich in ω−3 PUFAs helped prevent major depressive disorder (Lakshimi and Kavitha 2023). Additionally, ω−3 PUFA supplements enhanced the body’s ability to combat free radical harm, decreased lipid peroxidation (LPO), and prevented brain tissue aging (Kerdiles et al. 2017).

Recently, there has been an increase in the popularity of using naturally derived products to modulate MSG toxicity. Therefore, the present study aimed at further elucidating the mechanisms behind MSG-induced toxicity in the brain cortex of male rats. The study also focuses for the first time on exploring the protective effect of ω−3 PUFAs on MSG-induced neurotoxicity by monitoring the redox state, inflammatory markers, neurotransmitters release and their catabolizing enzymes, apoptosis, as well immunohistochemical and histopathological examinations.

## Materials and methods

### Chemicals

Monosodium glutamate (C_5_H_8_NO_4_Na, anhydrous, purity 99%) was purchased from Loba Chemie for laboratory reagents and fine chemicals (India). ω−3 PUFAs (Natrol^®^ Omega-3 fish oil) were obtained from Natrol LLC, Chatsworth (CA 91311, USA). Bovine serum albumin, glutathione (GSH), 2- thiobarbituric acid, NADPH, pyrogallol, 1,1,3,3-tetraethoxypropane, 5, 5′dithiobis-(2- nitrobenzoic acid) (DTNB) were obtained from Sigma-Aldrich (St. Louis, MO, USA). All other chemicals used in the experiment were of high analytical quality.

### Animals and ethical approval

The study protocol and all animal handling methods followed the guidelines of Animal Care and Use for Scientific Purposes. The approval for the experiment was granted by the Institutional Animal Ethics Committee at Alexandria University (Approval number AU04190525101). Forty male adult Wistar rats, weighing 170–190 g and aged 3–4 months, were acquired from the animal house at the Faculty of Medicine, Alexandria University. They were housed in stainless-steel cages and maintained under controlled conditions (relative humidity of 50–60%, temperature 25˚C, and a 12 h light/dark cycle) with access to a normal diet and tap water freely for two weeks of acclimatization.

### Experimental design and sampling

After the acclimatization, four groups of rats (*n* = 10) were allocated: Control group received saline solution, ω−3 PUFAs group received 400 mg/kg ω−3 PUFAs (Zararsiz et al. 2011), MSG group administered 4 mg/g/day MSG dissolved in saline solution (Wuyt et al. 2023), and MSG plus ω−3 PUFAs group treated with both MSG and ω−3 PUFAs (4 mg/g/day and 400 mg/kg, respectively). The dose of MSG (4 mg/g) was selected to be higher than the no observed adverse effect level (NOAEL) (3200 mg/kg/day) considering the abusive and uncontrolled consumption of MSG in food. For ω−3 PUFAs, each capsule of Natrol^®^ Omega-3 fish oil contained 1000 mg fish oil (EPA 180 mg and DHA 120 mg). The contents of the capsules were properly emptied and dissolved in corn oil equivalent to 72 mg/ml. The dose of ω−3 PUFAs was selected based on the efficacy of this dose in protecting the brain against formaldehyde-induced damage. MSG and ω−3 PUFAs were administered orally by gastric gavage each day for 4 weeks at consistent times. By the end of the treatment protocol, rats were fasted for 12 h and anesthetized using ketamine (40 mg/kg) before subjecting to euthanization. Rats from the control and treated groups were sacrificed by decapitation and were dissected for isolation of brain cortex from each rat. Brain cortices were collected from 5 rats in each group, fixed in 10% formalin, and processed routinely for histological and immunohistochemical examinations. The cortices collected from the other 5 rats in each group were cleaned, washed with ice-cold saline solution, and homogenized (10% w/v) separately in 50 mM cold potassium phosphate (pH 7.4). The homogenate was centrifuged at 12,000 rpm at 4ºC for 15 min. The supernatants were collected and stored at −80 ºC for further analysis.

### Determination of redox status indices in the brain cortex

LPO level (as malondialdehyde, MDA) was determined by spectrophotometric quantitation of the secondary MDA product after LPO by thiobarbituric acid (TBA), the absorbance was determined at 532 nm (Ohkawa et al. 1979). It is expressed as nmol/g tissue. Nitric oxide (NO) level. expressed as µmol/g tissue was determined in the cortex homogenates using a colorimetric method based on the Griess reaction according to the method described by Montogomery and Dymock (1962). Reduced glutathione (GSH) level, expressed as mmol/g tissue, was determined in the brain cortex using Elman’s reagent (5, 5′-Dithiobis [2-nitrobenzoic acid]) as outlined by Beutler and Kelly (1963). The activity of superoxide dismutase (SOD) was determined according to the protocol of Nishikimi et al. (1972). This assay depends on the ability of SOD to inhibit the phenazine methosulphate-mediated reduction of nitro blue tetrazolium dye. Catalase (CAT) activity was determined by monitoring the decline in absorbance due to the degradation of hydrogen peroxide (H_2_O_2_) at 240 nm as outlined by Aebi (1984). The activity of SOD and CAT are expressed as U/g tissue.

### Determination of inflammatory mediators in the brain cortex

Tumor necrosis factor-α (TNF-α) and interleukin-1β (IL-1β) were quantitively measured in the brain cortex using the ELISA technique following the manufacturer’s instructions TNF-α: Catalog No. RAB-0480, Sigma-Aldrich chemical company, USA. IL-1β: Catalog No. RAB-0278, Sigma-Aldrich chemical company, USA. They are expressed as pg/g tissue.

### Determination of neurochemical parameters in the brain cortex

Monoamine oxidase (MAO) activity was determined kinetically as described by Sandler et al. (1981) using p-tyramine hydrochloride as a substrate. The activity of acetylcholine esterase (AchE), and the levels of dopamine (DA) and serotonin (5-HT) in the cortex were assayed using a sandwich solid-phase ELISA kit as described in the manufacturer’s instruction. AchE: Catalog No. E-BC-K174-M, Elabscience, USA; DA: Catalog No. MBS-262,606, My BioSource, San Diego, USA; 5-HT: Catalog No. MBS-725,497, My BioSource, San Diego, USA.

### Histopathological examination

The frontal cortex from both hemispheres was carefully isolated from the whole brain according to the guidelines of the stereotaxic rat brain atlas (Paxinos and Watson 2007). It was then fixed in 10% formalin, transferred to xylol, and implanted in paraffin wax. The paraffin-embedded tissue was sectioned using rotary microtome at 5 μm thick coronal sections. The sections were stained with hematoxylin-eosin (H&E) for the histopathological observations (Hussein et al. 2017). Another set of sections was processed to assess the immunoreactivity of caspase-3.

### Immunoreactivity of caspase-3

Avidin-biotin complex technique was used for immunohistochemical examinations of caspase-3 in the cerebral cortex (Essawy et al. 2023). Paraffin sections from the cerebral cortex were prepared for immunohistochemical investigation of caspase-3 using avidin-biotin complex (ABC) technique. The polyclonal antibody used are anti-caspase-3 antibody (ab4051, Abcam, Inc). Sections of 5 μm thick were deparaffinized, rehydrated, and immersed in phosphate buffered saline (PBS). Endogenous peroxidase was inhibited by incubating the slides in 3% hydrogen peroxide for 10 min at room temperature before washing the slides in PBS. Sections were incubated with avidin-biotin blocking reagent for 30 min to block endogenous avidin and biotin binding sites. After that, the sections were incubated with primary antibody against caspase-3 for 60 min at room temperature. Next, sections were incubated with biotinylated secondary antibody followed by avidin–HRP-conjugated solution according to the manufacturer’s instructions. Afterward, the sections were treated with 3,3′-Diaminobenzidine to visualize the bound antibodies, rinsed in PBS for 2 min, and counterstained and differentiated in hematoxylin. After counterstaining, the sections were examined under the microscope to observe caspase-3 positive neurons and record the total number of positive cells. The intensity of positive immunolabeling for caspase-3 was quantitatively assessed in five fields from each section utilizing the Image J analysis software (version 5.1).

### Statistical analysis

Findings are shown as mean ± standard error of the mean (SEM). The statistical analysis was conducted with SPSS software (version 20). The data distribution’s normality was confirmed through the Shapiro-Wilk test. A one-way ANOVA was employed to compare the groups’ statistical differences and Tukey’s test was used for pairwise mean comparisons. The significant difference falls below the p-value of 0.05. The graphs were generated using GraphPad Prism software (version 6.01).

## Results

### ω−3 PUFAs ameliorated MSG-induced changes in the redox state of the brain cortex

**Fig. 1:**
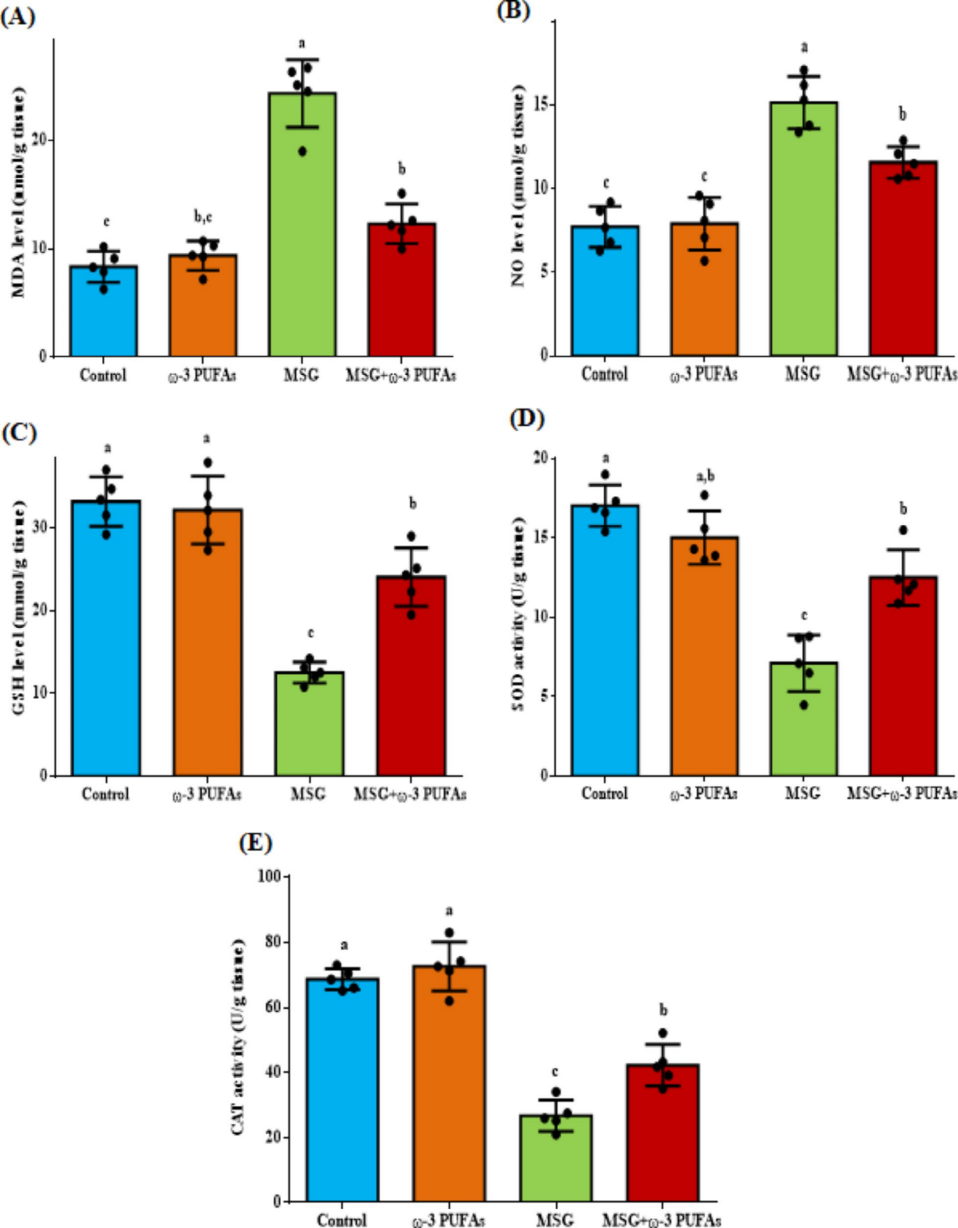
Effect of ω−3 PUFAs on MSG-induced changes in the redox status markers in rat’s cortical region. Groups were evaluated for their levels of MDA (**A**); NO (**B**); GSH (**C**); SOD (**D**); and CAT (**E**). Data are presented as mean ± SEM (*n* = 5). Significant differences (*p* < 0.05) between groups were indicated by different letters above the error bars. Columns with similar letters (a-c) indicate no significant differences. Abbreviations: CAT, catalase; GSH, reduced glutathione; MDA, malondialdehyde; MSG, monosodium glutamate; NO, nitric oxide; SOD, superoxide dismutase; ω−3 PUFAs, omega-3 polyunsaturated fatty acids

The brain cortex’s redox state was evaluated after the administration of MSG alone or together with ω−3 PUFAs (Fig. 1). There was a notable increase in the oxidative status in the brain cortex of the MSG-treated group, with MDA and NO levels showing an increase of 191% (F = 63.595, *P* < 0.001) and 95.9% (F = 33.826, *P* < 0.001), respectively, compared to the control rats. Conversely, the levels of antioxidants in the brain cortex of the MSG-treated group decreased significantly by 62.2% (F = 46.108, *P* < 0.001), 58.2% (F = 34.178, *P* < 0.001), and 61% (F = 73.748, *P* < 0.001) for GSH, SOD, and CAT (F = 73.748, *P* < 0.001), respectively compared to the control group. Supplementing of ω−3 PUFAs greatly improved the disturbance in the oxidative/antioxidative balance caused by MSG in the brain cortex, in comparison to the MSG group. There were no notable differences in MDA, NO, GSH, SOD, and CAT levels in rats given only ω−3 PUFA compared to the control group.

### ω−3 PUFAs attenuated MSG-induced neuroinflammation in the brain cortex

**Fig. 2:**
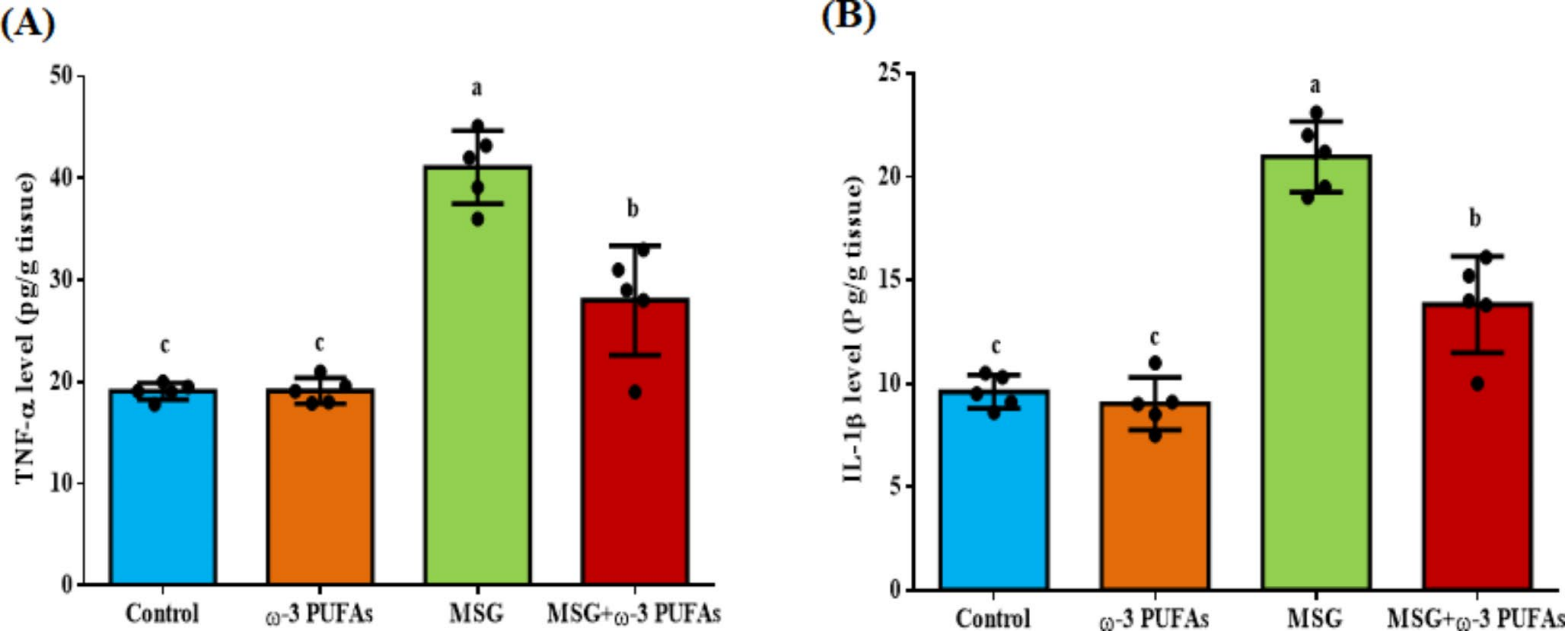
Changes in the inflammatory mediators in the brain cortical region of rats treated with MSG alone or in combination with ω−3 PUFAs. The levels of TNF-α (**A**); and IL-1β (**B**) were assessed in the brain cortex. Data are presented as mean ± SEM (*n* = 5). Bars with different letters (a-c) are significantly different (*p*<0.05). Abbreviations: IL-1β, interleukin 1β; MSG, monosodium glutamate; TNF-α; tumor necrosis factor-α; ω−3 PUFAs, omega-3 polyunsaturated fatty acids

According to Fig. 2, the treatment of MSG induced inflammation in the brain cortex of rats by causing a significant rise in levels of inflammatory mediators. Levels of TNF-α and IL-1β increased by 115.3% (F = 48.969, *P* < 0.001) and 118.35% (F = 57.079, *P* < 0.001), respectively compared to the control group. Administration of ω−3 PUFAs significantly reduced the inflammation triggered by MSG in the brain’s cortical region. The levels of TNF-α and IL-1β diminished by 31.8% and 34.1%, respectively, compared to MSG-treated rats. No notable change in levels of TNF-α and IL-1β was detected in rats treated solely with ω−3 PUFAs as compared to the control group.

### ω−3 PUFAs improve the neurochemical parameters in the brain cortex

**Fig. 3:**
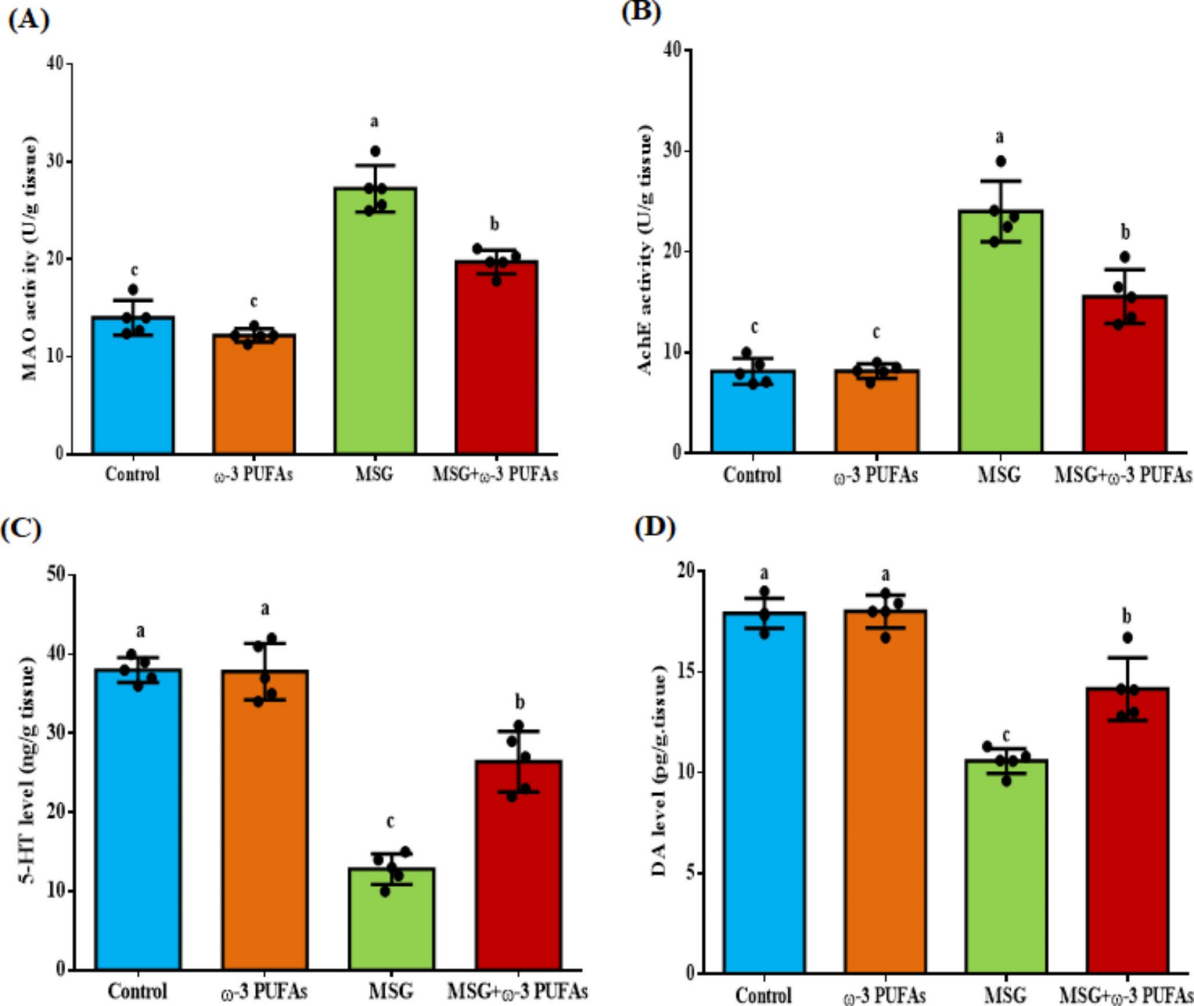
Neurochemical markers in the cortical region of rats treated with MSG alone or together with ω−3 PUFAs. Activities of MAO (**A**), AchE (**B**), and levels of 5-HT (**C**), and DA (**D**) were assessed. Values are presented as the mean ± SEM (*n* = 5). Bars with different letters (a-c) are significantly different (*p*<0.05). Abbreviations: AchE, acetylcholinesterase; DA, dopamine; MAO, monoamine oxidase; ω−3 PUFAs, omega-3 polyunsaturated fatty acids; 5-HT, serotonin

As shown in Fig. 3, MSG significantly increased MAO and AchE activities in the brain cortex by 94.6% (F = 51.266, *P* < 0.001) and 195.1% (F = 62.121, *P* < 0.001), respectively compared to the control rats. Conversely, levels of 5-HT and DA decreased significantly by 66.3% (F = 84.548, *P* < 0.001), and 40.9% (F = 37.481, *P* < 0.001), respectively in the MSG-treated group compared to the control group. Rats co-administered ω−3 PUFAs and MSG showed a significant improvement in the levels of these neurochemical indices in the brain cortex compared to the MSG group. Notably, no marked change was found in MAO, AchE, 5-HT, and DA in the cortex of rats treated with ω−3 PUFAs alone when compared to the control group.

### ω−3 PUFAs inhibited MSG-induced apoptosis

**Fig. 4:**
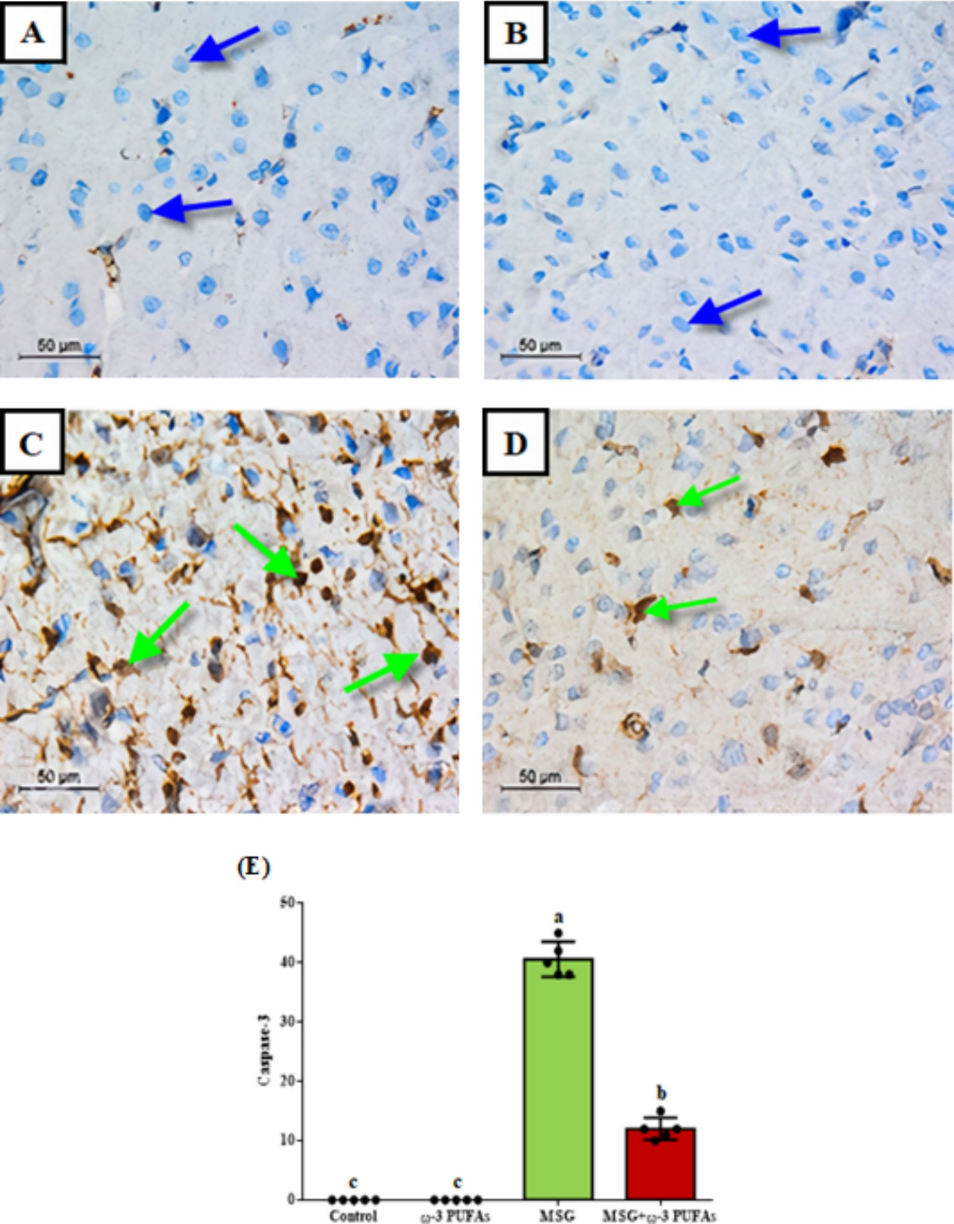
Photomicrographs of sections in rats’ cerebral cortex showing immunoreactivity to caspase 3. Sections of the control (**A**) and ω−3 PUFAs (**B**) groups show a negative immunostaining (blue arrow). Section from MSG-treated group (**C**) shows multiple caspase-3 positive neuronal cells (green arrow). Section from MSG plus ω−3 PUFAs group (**D**) shows a decline in the number of neuronal cells with positive staining (green arrow) (IHC, x400). (**E**) shows the number of cells immunopositive for caspase, data are presented as the mean ± SEM (*n* = 5). Bars with different letters (a-c) are significantly different (*p*<0.05). Abbreviations: MSG, monosodium glutamate; ω−3 PUFAs, omega-3 polyunsaturated fatty acids

Figure 4 shows the immunohistochemical analysis of caspase-3 in various experimental groups. Both control and ω−3 PUFAs alone groups displayed minimal immunostaining, indicating a lack of significant change in caspase-3 expression in both groups. However, the strong immunostaining in rats treated with MSG indicates a marked increase in caspase-3 expression compared to the control group, with a significance level at *p* < 0.001 (F = 596.569). Combining MSG with ω−3 PUFAs led to a marked decrease in caspase-3 expression compared to the group treated with only MSG. A decrease in immunostaining indicated a lowered caspase-3 expression in the MSG plus ω−3 PUFAs group relative to the MSG group.

### ω−3 PUFAs attenuated MSG-induced histological alterations in the brain cortex

**Fig. 5:**
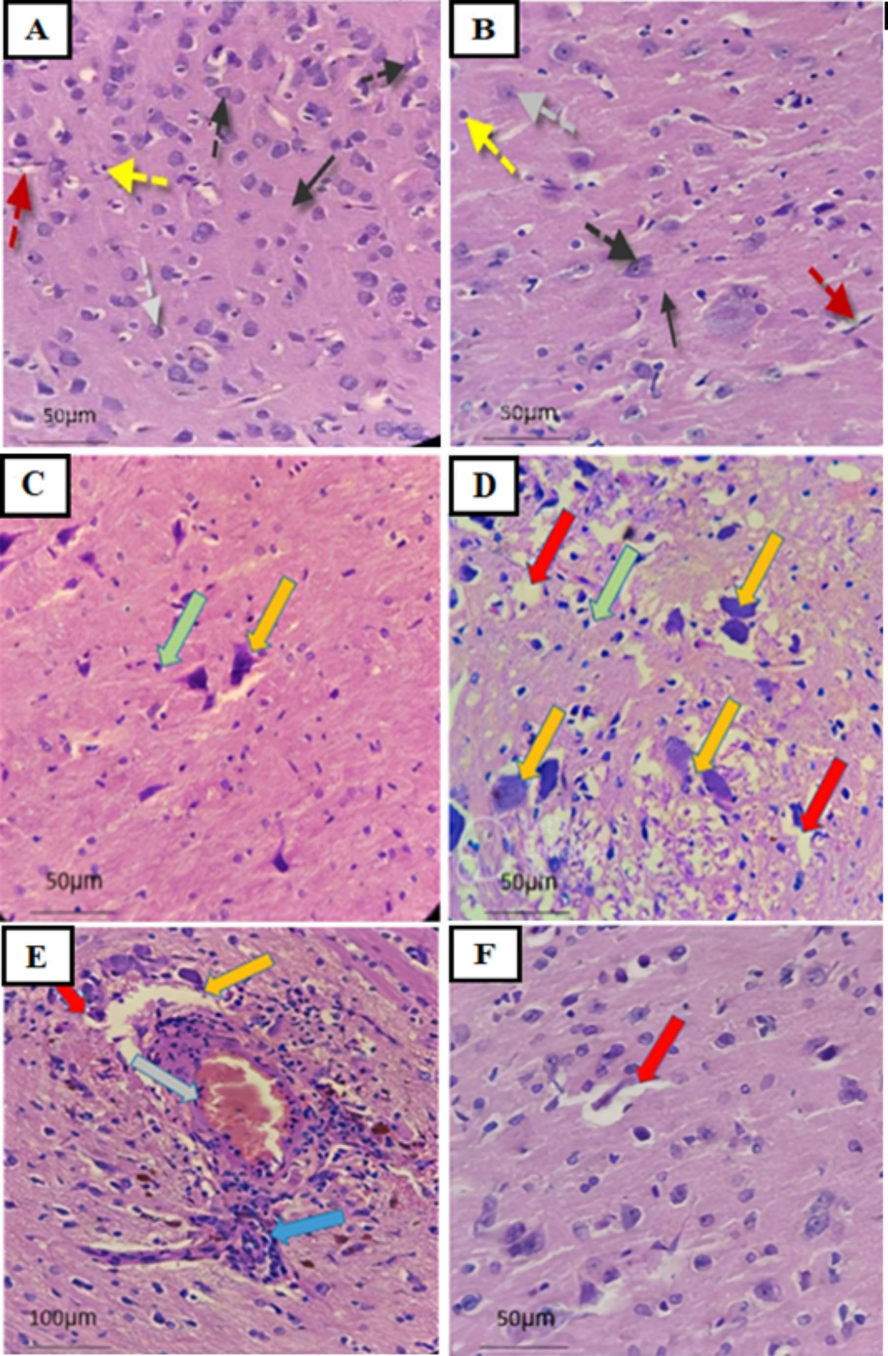
Photomicrographs of the brain cortical region from the control (**A**) and ω−3 PUFAs (**B**) groups, respectively showing normal architecture with normal neuropil (black arrow), normal granule cell with pale open face nucleus (gray dotted arrow), normal pyramidal cell (black dotted arrow), blood vessel (red dotted arrow), and glial cell (yellow dotted arrow). Photomicrographs (**C**-**E**) represent sections of brain cortex of MSG-treated rats, showing severe inflammatory infiltrate (blue arrow), marked edema (red arrow), degenerated neurons (yellow arrow), apoptotic cells (green arrow), and vascular congestion (grey arrow). Photomicrograph (**F**) represents sections of the brain cortex from MSG plus ω−3 PUFAs, showing mild edema (red arrow), rare apoptotic cells with subsided inflammation, neuron degeneration, and vascular congestion. H&E, scale bar: 50 μm (**A**, **B**, **C**, **D**, **F**) and 100 μm (**E**)

The outcomes of histological examination of the cerebral cortex in various experimental groups are depicted in Fig. 5. Photomicrographs from the control and ω−3 PUFAs groups (5 A&B) showed normal cortical histological features: normal neuropil, granule cells with pale open face nucleus, pyramidal cell, blood vessel, no vacuolation, and glial cell. In contrast, photomicrographs of the cerebral cortex from the MSG-treated group (5 C-E) displayed severe inflammatory infiltrate, noticeable edema, severe neuronal degeneration, apoptotic cells, vacuolated neuropil, and increased vascular congestion. In the MSG plus ω−3 PUFAs group, the cerebral cortex displayed mild swelling, few apoptotic cells, and subsided inflammation, nerve cell damage, and vascular congestion (Fig. 5F).

## Discussion

Monosodium glutamate (MSG) is a worldwide flavor enhancer. Even with concerns about its safety, MSG is still widely consumed. Dysregulation of glutamate signaling in the central nervous system is linked to many neurological disorders due to its role as an important excitatory neurotransmitter, leading to excitotoxicity and neuronal cell death. This study looked into the potential protective role of ω−3 PUFAs in combating MSG-induced neurological toxicity in the brain cortex of an experimental rat model. Findings supported the toxic effect of MSG, with notable rises in oxidative indicators (MDA and NO), inflammation markers (TNF-α and IL-1β), neurotransmitters-catabolizing enzymes (MAO and AChE), and apoptotic neural cell death (caspase-3). Conversely, the MSG-treated group showed a decrease in the antioxidants (GSH, CAT, and SOD) and the neurotransmitters level (5-HT and DA). In addition, MSG caused histopathological changes in the brain cortical tissue structure that align with the findings of biochemical and immunohistochemical analysis. Supplementation of ω−3 PUFAs greatly improved the biochemical, immunohistochemical, and histopathological alterations caused by MSG in the brain cortex.

Increased metabolic activity and reduced antioxidant capacity in brain cells are key factors in neurodegenerative disorders, making oxidative stress a defining feature. Based on the development of numerous neurodegenerative diseases, certain brain areas including the cortex, are vulnerable to oxidative stress (Saleem et al. 2020). Excessive formation of free radicals can result in the oxidation of membranes and nucleic acids, ultimately leading to apoptosis (Essawy et al. 2018). Additionally, an excess of free radicals can activate pathways involved in inflammation, potentially leading to impairment of the blood-brain barrier (Olufunmilayo et al. 2023). The current study demonstrated that MSG treatment caused oxidative damage in the cerebral cortex by changing the redox state, as shown by increased MDA and NO levels alongside decreased GSH levels and SOD and CAT activities. Our findings align with Kesherwani et al. (2024) showing that administration of MSG caused a notable rise in MDA and NO levels, while reducing GSH levels and the activities of SOD and CAT in the brain of male rats, indicating oxidative stress. Albrakati (2022) also found comparable findings. Excessive glutamate can lead to the buildup of ROS and LPO, which could be critical factors in glutamate-induced neuronal damage (AL-Nasser et al. 2022). The rise in NO levels found in our research could be caused by mitochondrial dysfunction and elevated cytokine production, leading to the activation of inducible nitric oxide synthase expression (Albrakati 2022). Oxidative stress caused by MSG can lead to decreased expression of nuclear factor erythroid 2 related factor 2 (Nrf2), as well as lower levels of antioxidant defense molecules like glutathione, heme oxygenase-1, SOD, and CAT (Liao et al. 2020). The reduction in GSH level, which indicates tissue degeneration, could result from its use to neutralize the surplus reactive oxygen species (ROS) in rats treated with MSG (Mohebbati et al. 2017). Moreover, the overproduction of ROS in animals treated with MSG could hinder neuronal cells’ capability to metabolize extracellular glutamate, causing a decrease in GSH levels (Singh and Panda 2023). The significant suppression of SOD activity in our study verifies the oxidative stress status induced by MSG. Moreover, ROS can stimulate microglia in the brain, leading to the release of pro-inflammatory cytokines, thus increasing oxidative stress (Rius-Pérez et al. 2020). Results of our study revealed that administration of ω−3 PUFAs attenuated oxidative stress and refurbished the antioxidant capacity in the cortical region of the MSG group. The protective impact of ω−3 PUFAs on MSG-induced oxidative stress could be due to their significant antioxidant activity in replenishing the disrupted endogenous antioxidants. EPA, a member of ω−3 PUFAs, has been shown to stabilize the membrane structure, decrease ROS generation, ameliorate LPO, and hamper the discharge of IL-1 and TNF-α (Oppedisano et al. 2020). ω−3 PUFAs are a source of naturally occurring antioxidants that can protect neuronal cells and make them less susceptible to LPO (Kapoor et al. 2021).

Neuroinflammation is well known to play a significant role in the onset and advancement of many neurological disorders (Essawy et al. 2024). It is mediated through the secretion of various pro-inflammatory cytokines with the involvement of ROS. Our study found that giving MSG increased levels of TNF-α and IL-1β in the cerebral cortex, indicating the incidence of neuroinflammation. MSG exhibits pro-oxidative effects through excess ROS production, causing the production of inflammatory cytokines like TNF-α and IL-6 (Das et al. 2024). ROS can also disrupt mitochondrial function, resulting in reduced ATP synthesis and enhanced ROS production (Missiroli et al. 2020). Moreover, ROS can disrupt the blood-brain barrier, enabling external inflammatory and immune cells to enter the brain which enhances the brain’s inflammatory reaction and heightens damage to neurons (Song et al. 2020). MSG may cause inflammation in the brain by activating specific receptors in brain cells which promote translocation of the nuclear factor-κB (NF-κB) to the nucleus and induce the expression of IL-1β and TNF-α (Abu-Elfotuh et al. 2022). Concurrent administration of ω−3 PUFAs in our study markedly attenuated the inflammation induced by MSG in the brain cortical area. Studies showed that ω−3 PUFAs protected the blood-brain barrier and reduced inflammation in the brain cells of a rat model of Alzheimer’s disease (Xie et al. 2020). ω−3 PUFAs have been reported to reduce the activation of NF-κB induced by lipopolysaccharide, resulting in decreased production of IL-1β and TNF-a, as well as chemokines (Lu et al. 2013). Moreover, ω−3 PUFAs and their active metabolites can reduce the expression of inflammatory genes and promote the production of lipid mediators that help resolve inflammation (Oppedisano et al. 2020).

Apoptosis is a vital physiological process necessary for the proper development of the nervous system with caspase-3 recognized as a pivotal player in the apoptotic pathway (Nguyen et al. 2021). Nonetheless, exposure to neurotoxic substances can cause harmful effects on neuronal cells, including increased ROS production, disrupted calcium balance, and initiation of mitochondrial permeability transition due to mitochondrial dysfunction. These occurrences trigger the cytochrome C release, subsequently activating caspase-3 and causing a gradual rise in neuronal cell mortality (Chen et al. 2022). In our study, there is a rise in caspase-3 immunoreactivity in neuronal cells of the brain cortex of rats treated with MSG based on immunohistochemical analysis. Reports from Abu-Elfotuh et al. (2022) and Albrakati (2022) showed a comparable rise in caspase-3 expression in rat brains. MSG-induced oxidative stress, inflammation, and DNA damage might lead to elevated caspase-3 expression and worsening apoptosis-induced death (Matés et al. 2012). A study connected immunohistochemical changes and brain injury to oxidative stress, glutamate levels, and AChE/caspase-3 activity (Onaolapo et al. 2019). Increased levels of glutamate in the synapse may result in overstimulation and harm to nerve cells through excitotoxicity. Moreover, excessive activation of glutamate receptors disturbs the balance of calcium levels by enhancing the entry of calcium ions and their release from cellular stores. This leads to the activation of hydrolytic enzymes and contributes to the degeneration and death of nerve cells through apoptosis (Dong et al. 2009). The increased calcium influx and the destruction of the internal mitochondrial membrane potential cause unregulated mitochondrial permeability of the pores to apoptotic markers (Kanki et al. 2004). Coadministration of ω−3 PUFAs and MSG decreased caspase-3 levels compared to the group intoxicated with MSG alone. ω−3 PUFAs have been reported to inhibit the shift of microglial cells to a pro-inflammatory state and protect neuronal function after a traumatic brain injury (Lin et al. 2023). Additionally, a significant link was found between the administration of DHA and the prevention of cell death in M17 cells, resulting in a decrease of more than 66% in active caspase-3 protein level in treated cells compared to untreated cells (Suphioglu et al. 2010). The reduction in caspase-3 levels could be linked to the protective function of ω−3 PUFAs in preventing oxidative damage and neuronal cell death.

Increased extracellular glutamate causes aberrant synaptic signaling leading to neuronal excitotoxicity, often causing nerve cell death due to excessive activation of glutamate receptors (Iovino et al. 2020). Studies on MSG in different areas of the brain have consistently shown its harmful effect on neurotransmission, cognition, and behavior (Gbadamosi et al. 2024). Acetylcholine (ACh) plays a crucial role in cholinergic neurotransmission, its level is influenced by the enzyme acetylcholine esterase (AChE). Higher AChE activity is anticipated to lower ACh levels and disrupt the cholinergic system, leading to neurodegenerative diseases. DA and 5-HT are essential neurotransmitters that control numerous brain functions. Reduced concentrations of DA and 5-HT are believed to be involved in depression, anxiety, and neurodegenerative diseases (Essawy et al. 2017). MAO is an enzyme that partially breaks down neuroactive monoamines, leading to a decrease in their levels. In the present study, giving MSG led to a rise in the activities of AChE and MAO alongside a decline in DA and 5-HT levels. These findings align with Albrakati (2022) and Abdelhamid et al. (2024) who found higher AChE activity and lower levels of DA and 5-HT in the rat brain after being treated with MSG. The rise in AChE activity may be associated with oxidative stress, DNA damage, and disruption of the respiratory complex in the brain (Wong et al. 2020). It is worth to mention that Hussein et al. (2017) reported an inhibition in the activity of AchE in the cerebral cortex of MSG-treated rats. The decrease in levels of DA and 5-HT could be linked to the heightened activity of their breaking-down enzyme, MAO. Heightened glutamate levels in the brains of rats treated with MSG significantly raised NO levels, leading to mitochondrial dysfunction and DA-related neurotoxic effects. Additionally, the significant decrease in levels of DA and 5-HT could be a result of damage to the brain cortex, leading to disrupted neurotransmitter release due to disturbances in calcium balance caused by MSG (Ankul et al. 2023). Concurrent administration of ω−3 PUFAs with MSG reduced MSG-induced perturbations in the neurochemical parameters by lowering AChE and MAO activities while increasing levels of DA and 5-HT. Our findings support Abdou et al. (2017) that ω−3 fatty acids lowered AChE activity and raised the level of DA in the brain of β-cyfluthrin-treated rats. The elevated levels of DA and 5-HT in rat brains may be attributed to the neuroprotective and neurotransmitter-regulating properties of ω−3 PUFAs. Moreover, ω−3 fatty acids may lower LPO, a crucial factor in preserving AChE function and protecting the cholinergic system in the brain. Insufficient consumption of ω−3 nutrients, especially DHA, significantly affects the dopaminergic, serotoninergic, and glutamatergic systems (Moreira et al. 2010).

The neurotoxic effect of MSG was confirmed through analysis of histopathological changes in the brain cortex. The observations revealed severe neuronal degeneration, as evidenced by inflammatory infiltration, significant edema, degenerated neurons, apoptotic cells, and vascular congestion. These findings are consistent with Kesherwani et al. (2024) and Abdelhamid et al. (2024) who found significant neurodegeneration in the cerebral cortex even at low MSG intake. MSG can lead to neurodegeneration in animals by inducing damage to brain structure and degeneration of neurons. Due to its elevated PUFA levels, the brain is highly prone to peroxidation of its membrane lipids (Fu et al. 2022). Therefore, the histopathological changes induced by MSG in the present study can be linked to oxidative harm and neuronal cell death. The increased apoptotic bodies and neuronal damage in the brain cortex of MSG-treated rats could be the reason for the histopathological changes observed, as shown by the elevated immunoreactivity of caspase-3 protein. On the contrary, MSG-induced neurotoxicity was notably improved by ω−3 PUFAs supplementation, providing additional evidence of its neuroprotective impact on MSG-induced neurotoxicity. The ability of ω−3 PUFAs to scavenge radicals, act as an antioxidant, reduce inflammation, and suppress apoptosis could be the reason for the observed improvement in the cerebral cortex tissue.

## Conclusion

The outcomes of the current study showed biochemical, neurochemical, immunohistochemical, and histopathological changes in the rat’s cortical region with the administration of MSG. It induced oxidative stress, disrupted the neurotransmitters, and enhanced inflammation and apoptosis, in addition to histopathological deterioration in the brain cortex architecture. The results also provided evidence for the neuroprotective effect of ω−3 PUFAs against MSG-induced neurotoxicity. The protective effect offered by ω−3 PUFAs is attributed largely to the ability to scavenge radicals and act as an antioxidant, reduce inflammation by inhibiting inflammatory mediators, and suppress apoptosis by downregulating caspase-3. These findings recommend restricting the consumption of MSG as a flavor enhancer and emphasize the beneficial effect of ω−3 PUFAs supplement to counteract MSG neurotoxicity. A further study is recommended to have a more thorough understanding and justify the exact molecular mechanism(s) by which ω−3 PUFAs provide their protective effect.

## Data Availability

No datasets were generated or analysed during the current study.
